# General Commentary: Alendronate Use and Risk of Type 2 Diabetes: A Nationwide Nested Case-Control Study

**DOI:** 10.3389/fendo.2022.893735

**Published:** 2022-06-06

**Authors:** Burkhard Muche

**Affiliations:** Department of Rheumatology and Clinical Immunology, Charité Universitaetsmedizin, Berlin, Germany

**Keywords:** diabetes mellitus, prevention, alendronate, retrospective, osteocalcin

## Background

Osteoporosis and diabetes mellitus are common metabolic diseases. Interventions to prevent either (or to kill two birds with one stone) seem worthwhile.

## Population-Based Research

The current study (1) was based on nationwide databases in Denmark. The authors defined 163,588 subjects with a diagnosis of diabetes mellitus and matching controls. There were many imbalances, e.g., smoking, glucocorticoid use, and comorbidities. The researchers looked into prior Alendronate use since 1998. A protective effect (HR = 0.64) against type 2 diabetes was calculated, as well as some dose dependency. The results in the work of Viggers et al. might have been confounded: There is well-known gastric intolerance to oral bisphosphonates, which would keep several patients from taking more than a single or limited dose of the drug. Some patients might have never taken any pill after reading about possible side effects in the package insert, and no individual intake evaluation was possible. The reported dose effect (with longer ones taking Alendronate, which lowers diabetes risk), which should support the concept that Alendronate protects against diabetes, is modest and overexpressed, as shown in Figure 1 of the paper by Viggers et al. Alendronate ≤ 6 months, however, reduced diabetes risk by 30% (HR = 0.70), with the “most effective” time frame of 4.0-5.9 years adding just 14.7% (HR = 0.55). A year-long effect on glucose metabolism from possibly very limited medication exposure in the first weeks or months with quite little improvement in long-term treatment groups seems relatively implausible.

Interestingly, similar information was published by the co-author Vestergaard (2011) (2). In that historic paper, he did a different data selection, comparing 103,562 users of osteoporosis drugs matched to controls (not taking osteoporosis drugs). Dose-dependent protection to develop diabetes by use of Alendronate (HR = 0.71) was reported. The strongest benefit against developing diabetes mellitus was found with the selective estrogen receptor modulator Raloxifene (HR = 0.46), but this fact did not make it to the title of the paper. Another interesting point is that, in the control group (without osteoporosis medication), there were 4.1% with diabetes at baseline, compared to 3.5% in the osteoporosis group!

Despite an impressively high number of participants and best attempts to adjust for confounders in those studies, results are only associations and critical information, e.g., body mass index, was missing (subjects with higher BMI have a higher incidence of DM but have lower incidence of osteoporosis).

A study by Toulis et al. (3) using UK databases also discussed this aspect by comparing 35,998 bisphosphonate users vs. 126,459 matched non-users with the advantage to include BMI into matching. They also reported an overall protection against diabetes, but curiously only in the long term (more than 2.5 years of exposure), while somewhat confusingly, an increase in their first quartile (1.0–2.5 years) was seen with an adjusted IRR of 1.67. They chose to exclude medication exposure of less than 1 year from analysis.

## Preclinical Trials

Osteocalcin is a possible connector between bone and glucose metabolism. Preclinical data are contradictory; e.g., Alendronate reduced osteocalcin and might improve glucose metabolism by that way in several cell culture experiments (4); on the other hand, there was an animal model with Alendronate-fed rats (5), reporting higher fasting blood glucose levels and worsened insulin sensitivity.

## Prospective Clinical Trials

A study by Hong et al. (6) in 84 osteoporotic patients starting risedronate (plus vitamin D) for 16 weeks did show the expected decline of about 50% in osteocalcin levels but a slightly increased (!) fasting plasma glucose (5.3–>5.5 mmol/L) without other changes in the glucose metabolism (insulin, HOMA-B, and HOMA-IR).

Evidence from RCTs with this topic is scarce. In 2019, Fard et al. (7) published a clinical trial in 80 non-diabetic postmenopausal women “with osteopenia” (but BMD was not provided). They suggested some benefit of Alendronate vs. placebo on glucose metabolism over 12 weeks: small reductions in the Alendronate-treated group in fasting glucose (between group difference 5.7 mg/dl = 0.3 mmol/L) and Hb1Ac (Δ 0.11%) and an improvement of HOMA insulin resistance were reported (−0.95 with Alendronate vs. −0.08 with placebo, but intergroup difference was not significant). There were imbalances at randomization: HbA1c and HOMA-IR were higher in placebo. A dropout rate of 25% in just 12 weeks’ study duration has to be considered, and no intention-to-treat (ITT) statistics was applied. A crossover of the placebo group would have been interesting.

Schwartz et al. (8) analyzed data of three large pivotal trials of osteoclast inhibitors (Alendronate, Zoledronic Acid, and Denosumab) in ~20,000 postmenopausal osteoporotic women. Despite being a *post hoc* evaluation, the advantages of this ITT analysis were as follows: substantial patient numbers; prospective and randomized population including clinical information such as compliance with medication, body mass index, and bone mineral density; and, of course, lab values. They summarized: “Antiresorptive therapy does not have a clinically important effect on fasting glucose, weight, or diabetes risk in postmenopausal women”.

## Conclusion

To conclude, the study by Schwartz et al. has the best database to draw the conclusion: There is no clinically relevant effect of bisphosphonates on glucose metabolism in humans, whatever preclinical models may suggest. Taking into account the mentioned literature, another hypothesis for future research is suggested: “Do osteoporosis patients have a statistically lower risk to develop diabetes mellitus compared to a non-osteoporotic population?”—getting diagnosed with osteoporosis might have at least one small benefit for the patient.

## Author Contributions

The author confirms being the sole contributor of this work and has approved it for publication.

## Funding

I acknowledge financial support from the Open Access Publication Fund of Charité – Universitätsmedizin Berlin and the German Research Foundation (DFG).

## Conflict of Interest

The author declares that the research was conducted in the absence of any commercial or financial relationships that could be construed as a potential conflict of interest.

## Publisher’s Note

All claims expressed in this article are solely those of the authors and do not necessarily represent those of their affiliated organizations, or those of the publisher, the editors and the reviewers. Any product that may be evaluated in this article, or claim that may be made by its manufacturer, is not guaranteed or endorsed by the publisher.
